# Flexor Tenosynovitis Caused by *Neisseria gonorrhea* Infection: Case Series, Literature Review, and Treatment Recommendations

**DOI:** 10.1055/a-1938-0837

**Published:** 2023-02-01

**Authors:** Nirbhay Jain, Sean Saadat, Mytien Goldberg

**Affiliations:** 1 Division of Plastic and Reconstructive Surgery, University of California, Los Angeles, California; 2 Division of Plastic and Reconstructive Surgery, Harbor-UCLA Medical Center, Los Angeles, California

**Keywords:** flexor tenosynovitis, gonorrhea, nonoperative management

## Abstract

*Neisseria gonorrhoeae* is the most common sexually transmitted disease in the world and is known to cause disseminated disease, most commonly tenosynovitis. Classically, gonorrhea-associated tenosynovitis presents with concomitant dermatitis and arthralgias, though this is not always the case. *N. gonorrhoeae* -related tenosynovitis has become more commonly seen by hand surgeons. To aid in management, we present three cases of gonorrhea-induced tenosynovitis spanning a range of presentations with variable treatments to demonstrate the variety of patients with this disease. Only one of our patients had a positive gonococcal screening test and no patient had purulent urethritis, the most common gonorrhea-related symptom. A separate patient had the classic triad of tenosynovitis, dermatitis, and arthralgias. Two patients underwent operative irrigation and debridement, and one was managed with anti-gonococcal antibiotics alone. Though gonorrhea is a rare cause of flexor tenosynovitis, it must always be on the differential for hand surgeons when they encounter this diagnosis. Taking an appropriate sexual history and performing routine screening tests can assist in the diagnosis, the prescription of appropriate antibiotics, and potentially avoiding an unnecessary operation.

*Neisseria gonorrhoeae* ( *N. gonorrhoeae* , gonorrhea) is a Gram-negative bacterium with a wide range of clinical presentations. 1 Most commonly, gonorrhea presents as a sexually transmitted infection (STI) with purulent urethritis and mucosal infections. It is the most common STI in young adults under mandatory reporting, with an incidence that increased 63% from 2014 to 600,000 new cases in 2019. 2

Disseminated gonococcal infection (DGI), however, is a rare complication, presenting in 0.5 to 3% of patients. DGI develops after the body's immune system fails to prevent the spread of the infection beyond mucosal surfaces. 3 Risk factors include biological female gender, especially during menstruation and in pregnancy, as well as an immunocompromised state, such complement disease (critical to clearing *Neisseria* infections), asplenia, systemic lupus erythematosus, or human immunodeficiency virus (HIV) infection.

DGI typically has one of two constellations of symptoms: the classic triad of tenosynovitis, dermatitis with pustular lesions, and polyarthralgia as discussed above, or suppurative arthritis limited to one joint. Tenosynovitis is the most common symptom of DGI and usually occurs in the upper and lower extremities but rarely is isolated in one joint and without other symptoms. 4 5 6 In most cases, constitutional symptoms such as fever and chills are commonly present. Patients typically develop tenosynovitis and arthralgia due to immune complex deposition leading to an inflammatory response. As bacteria do not infiltrate the joint or tendon, cultures tend to be negative and joints are not destroyed.

In this report, we present three recent cases of flexor tenosynovitis (FTS) at our institutions due to DGI without mucosal symptoms and without the traditional DGI triad, with a range of treatments demonstrating the various options of care, while also summarizing current literature.

## Cases

### Case 1

This patient was a 21-year-old female who presented with 3 days of pain in her right index finger, which spread dorsal to volar, with the finger becoming flexed. She denied injury to the hand or any constitutional symptoms, including dysuria, discharge, or arthralgia.

**Fig. 1:**
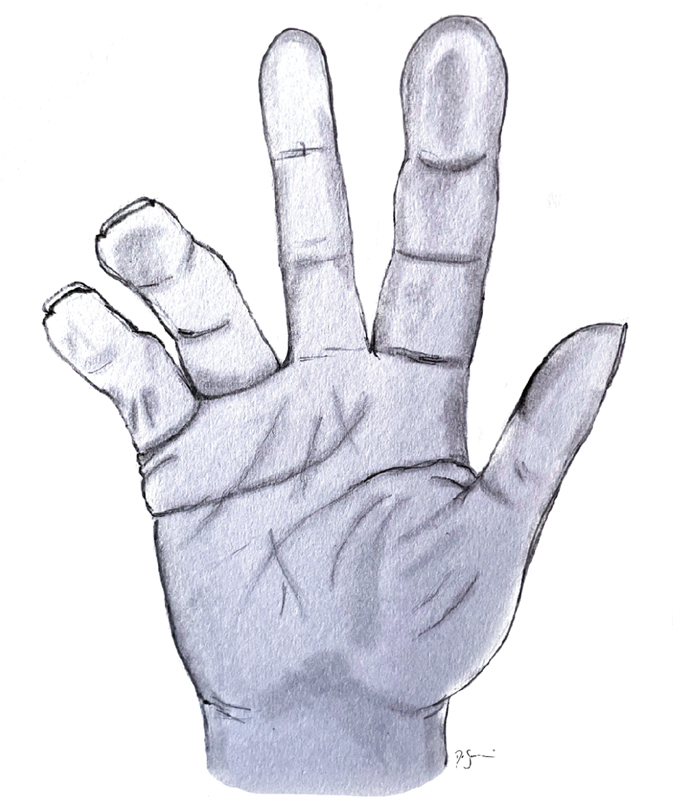
Schematic demonstrating our first patient. Notice the swollen right index finger with erythema.

On examination, she had a fever to 38.2°C. Her right index finger demonstrated all four Kanavel signs with intact sensation and perfusion to the fingertip. She had no triggering or nodularity along the flexor tendon ( Fig. 1 ). Imaging was unremarkable. Laboratory values were significant for a leukocytosis to 17.4. Blood cultures were not taken.

She was diagnosed with FTS and taken emergently to the operating room for a washout of her finger with return of purulent fluid, which was cultured.

Postoperatively, the patient was started on vancomycin and ceftriaxone. Intraoperative cultures resulted in multidrug-resistant *N. gonorrhoeae* , including to ceftriaxone. Infectious Diseases recommended double strength trimethoprim/sulfamethoxazole. On postoperative day 1, her symptoms had resolved and she was discharged on a 10-day course of oral antibiotics.

Urine gonorrhea testing was later found to be positive though she had no symptoms. Further immunocompetency and STI screening was negative except for herpes simplex virus. A comprehensive sexual history was not taken.

She was seen last on postoperative day 10 with complete resolution of symptoms.

### Case 2

This patient was a 32-year-old female who presented with 4 days of pain in her left ring finger. The patient has no prior history of trauma to affected finger. She developed fevers, chills, and arthralgias, but no dermatitis or urethritis.

On examination, she as well had all four Kanavel signs. She had intact sensation and perfusion to the fingertip. She had no triggering or nodularity along the flexor tendon. X-ray was normal. Laboratory values were significant for a white blood cell count of 21.

As gonorrhea was on the rise in our hospital system, DGI was high on our list of differential diagnosis due to the gender of the patient and lack of trauma to the affected digit. Thus, she was started on ceftriaxone without surgical intervention and improved greatly on hospital day 1. Laboratory work revealed blood cultures positive for *N. gonorrhoeae* and she was treated 2 weeks of intravenous ceftriaxone as definitive therapy. Further STI workup, including urine gonorrhea testing, was negative. A comprehensive sexual history was not taken. She was discharged on hospital day 4 and was last seen 2 weeks after discharge with complete resolution of symptoms.

### Case 3

This patient was a 25-year-old male who presented initially with 1 week of bilateral hand swelling, erythema, pain, and left knee pain. The pain began with a pustule in his right palm with pruritis but spread to diffuse bilateral hand pain, especially his left long finger. He denied any trauma to the area but does endorse sexual intercourse with an escort 10 days prior to presentation.

On examination, he had bilateral hand swelling, worse on the left than the right. His left hand was also significant for all Kanavel signs in the long finger. He was febrile to 38.7°C in the emergency room with a white blood cell count of 18.6. Blood cultures were negative.

He was initially admitted on intravenous vancomycin and piperacillin-tazobactam. Given the concern for FTS, he was taken to the operating room for a washout of his finger for presumed FTS. Both the left long finger and the right thumb were investigated with purulent drainage. Copious irrigation was performed and cultures were sent.

Postoperatively, the patient was started on vancomycin and ceftriaxone. Intraoperative cultures resulted in *N. gonorrhoeae* and Infectious Diseases was consulted. He was switched to intravenous ceftriaxone for 2 weeks. Further testing for gonorrhea, HIV, syphilis, and chlamydia were negative. Immune testing was negative

He was last seen 2 months after surgery with complete resolution of symptoms.

## Discussion

FTS is typically an emergent condition requiring immediate operative management due to possible loss of the tendon sheet and flexor mechanism. Typically, these infections are secondary to trauma and are caused by skin flora. Rarely, systemic infections can lead to seeding of the flexor sheath and resultant tenosynovitis.

DGIs are one systemic cause of tenosynovitis. These usually present with a classic triad of tenosynovitis, arthritis, and dermatitis after known mucosal gonococcal infection secondary to STIs, can have isolated tenosynovitis. If a diagnosis of tenosynovitis due to DGI is made, then operative management can be deferred, as most patients have full recovery on intravenous ceftriaxone alone. The danger with pursuing a nonoperative course, however, is the risk of missing a more destructive organism, especially since the diagnosis of DGI is often made after operative sampling of the offending organism. As evidenced by our cases above, patients still tend to undergo operative management because of questionable sources. Our one patient who avoided surgery did so because symptoms resolved after starting ceftriaxone in a high-incidence region for gonorrhea with positive blood cultures.

Making this situation more complicated is the varying presentations of the cases, as seen above. Only one of our three patients presented with the triad of dermatitis, arthralgia, and tenosynovitis and a risky sexual history that would be expected in DGI. Only one patient, separate from the patient with the triad of symptoms, had a positive urine gonococcal test. No patients had urethritis on exam. Compounding this is the lack of sexual history taken in the other two patients, limiting the ability to diagnose DGI. Despite no known trauma to the area, it was assumed that these patients had a standard FTS, not a more esoteric type, and potentially unnecessary operations were undertaken. Universal gonococcal screening is unlikely to assist in avoiding unnecessary surgeries, as only one patient had a positive gonococcal screening test.

Cases of gonococcal-induced tenosynovitis are on the rise, with case reports appearing in greater frequency over the past decade. 7 8 9 These reports universally describe operative management due to the concern for a more dangerous tenosynovitis infection and lack of confirmation of a gonococcal source. As seen in our patients, presentations can be widely variable, with some having the classic triad, some with urethritis, and some with isolated tenosynovitis, whether flexor or extensor.

Antibiotic regimens also vary—our cases describe both oral trimethoprim-sulfamethoxazole and intravenous ceftriaxone, while the prevailing trend in the literature is intravenous ceftriaxone for 2 weeks, which simply extends the traditional one-time intramuscular dose of ceftriaxone for gonorrhea. 10 Interestingly, a vancomycin and ampicillin-sulbactam combination helped treat our second patient, due to a pan-sensitive gonorrhea infection. Gonorrhea has previously demonstrated sensitivity to all β-lactams, tetracyclines, and macrolides, but resistance has built such that ceftriaxone is the only reliable treatment. The first case described was resistant to ceftriaxone, but luckily to trimethoprim/sulfamethoxazole, an alternative treatment, but superbugs have been found with no known treatment. 11 12

Given the complex and varied presentation of FTS due to DGI, it is important for any hand surgeon to consider DGI as a differential diagnosis when evaluating sexually active adolescents and young adults in absence of typical symptoms and trauma history. An estimated 2 million individuals in the United States have a gonococcal infection and cases are continuing to rise. The Centers for Disease Control continue to recommend yearly screening for gonorrhea in sexually active females with new or multiple sexual partners. 2 For any consult for tenosynovitis, it is prudent to take a comprehensive sexual history and screen patients with a urine gonococcal test if concerned to see if DGI is a likely cause of disease though a negative test does not rule out DGI. A full review of systems is also important to ensure dermatitis and arthralgias are not present. Though operative management is the gold standard in caring for these patients and remains the preferred option if there is any question on the diagnosis of DGI, if the suspicion for DGI is high enough based on history and exam without an obvious traumatic injury to the area, appropriate antibiotics, such as ceftriaxone, could be started and the patient can be observed to see if symptoms improve.

## Conclusion

DGI is a rare but not unfamiliar cause of FTS. Presentations can be variable though the disease can be managed nonoperatively if properly diagnosed in time. In this case series, we present three cases of gonococcal tenosynovitis with varying presentations and managements and review current literature on the topic. Gonococcal infections of this nature are increasing and should be in the differential for any hand surgeon evaluating sexually active patients with tenosynovitis. An appropriate sexual history and relevant screening tests, especially in patients without an obvious traumatic cause of FTS, should be ordered to guide antibiotic choices and determine if operative care is necessary or if antibiotic treatment alone could be trialed.
